# Cross-Domain Associations Between Motor Ability, Independent Exploration, and Large-Scale Spatial Navigation; Attention Deficit Hyperactivity Disorder, Williams Syndrome, and Typical Development

**DOI:** 10.3389/fnhum.2019.00225

**Published:** 2019-07-03

**Authors:** Emily K. Farran, Aislinn Bowler, Annette Karmiloff-Smith, Hana D’Souza, Leighanne Mayall, Elisabeth L. Hill

**Affiliations:** ^1^School of Psychology, University of Surrey, Guildford, United Kingdom; ^2^Wellcome Centre for Human Neuroimaging, University College London, London, United Kingdom; ^3^Department of Psychological Sciences, Birkbeck, University of London, London, United Kingdom; ^4^Department of Psychology, University of Cambridge, Cambridge, United Kingdom; ^5^Department of Psychology and Human Development, UCL Institute of Education, University College London, London, United Kingdom; ^6^Department of Psychology, Goldsmiths, University of London, London, United Kingdom

**Keywords:** attention deficit hyperactivity disorder, Williams syndrome, motor development, spatial cognition, navigation

## Abstract

In typical infants, the achievement of independent locomotion has a positive impact on the development of both small-scale and large-scale spatial cognition. Here we investigated whether this association between the motor and spatial domain: (1) persists into childhood and (2) is detrimental to the development of spatial cognition in individuals with motor deficits, namely, individuals with attention deficit hyperactivity disorder (ADHD) and individuals with Williams syndrome (WS). Despite evidence of a co-occurring motor impairment in many individuals with ADHD, little is known about the developmental consequences of this impairment. Individuals with WS demonstrate impaired motor and spatial competence, yet the relationship between these two impairments is unknown. Typically developing (TD) children (*N* = 71), individuals with ADHD (*N* = 51), and individuals with WS (*N* = 20) completed a battery of motor tasks, a measure of independent exploration, and a virtual reality spatial navigation task. Retrospective motor milestone data were collected for the ADHD and WS groups. Results demonstrated a relationship between fine motor ability and spatial navigation in the TD group, which could reflect the developmental impact of the ability to manually manipulate objects, on spatial knowledge. In contrast, no relationships between the motor and spatial domains were observed for the ADHD or WS groups. Indeed, while there was evidence of motor impairment in both groups, only the WS group demonstrated an impairment in large-scale spatial navigation. The motor-spatial relationship in the TD, but not the ADHD and WS groups, suggests that aspects of spatial cognition can develop via a developmental pathway which bypasses input from the motor domain.

## Introduction

The motor system is central to almost everything that we do. We use motor skills to interact socially, to produce language, in handwriting and in activities of daily living (e.g., eating, brushing hair). Motor activity is integral to early development; as motor ability develops, infants become more able to explore their environment and the objects within it. Here, we discuss the development of motor skills and their relationships to spatial cognition. For example, Clearfield (2004) demonstrated that infants who had been crawling or walking for more than 6 weeks were better able to use a landmark to find a goal location in a large octagonal space than infants with less crawling or walking experience. Furthermore, performance on the A-not-B task (Piaget, 1952), which has a large spatial component (alongside factors such as cognitive flexibility and object concept), has been linked to locomotor experience (Bertenthal et al., 1994). In this task, infants observe a toy being repeatedly hidden in one of two locations (A) and successfully find the toy. However, when they then observe the toy being hidden in location B, they perseveratively search in location A for the toy. This spatial error ceases to be made once infants have had sufficient crawling experience; for example, at 7.5 months, the length of time that an infant has been crawling or using a baby walker predicts their ability to solve this task (Bai and Bertenthal, 1992). This is thought to relate to the transition from body-centered spatial coding, to the ability to track landmarks and objects independent of the infant’s own (changing) location once crawling has begun.

The relationship between motor ability and spatial cognition is not just limited to gross motor abilities. Soska et al. (2010), for example, demonstrate an association between visual-manual exploration skills and 3D object perception. The authors found that, in 4.5- to 7.5-month-old infants, the motor skills that are required to change the viewpoint of an object (rotating, fingering, and transferring objects between hands while simultaneously looking at them) were predictive of their ability to determine the spatial properties of 3D objects accurately when viewed from a single viewpoint. This demonstrates that the development of visual-manual skills facilitates the generation of knowledge surrounding object properties.

Beyond infancy, little is known about the relationship between motor skills, motor experience, and spatial knowledge. Longitudinal evidence has demonstrated that age of walking, as well as exploration through locomotion at 20 months, are both related to performance on the Block Design task of the Wechsler scales (Wechsler, 1999), a measure of spatial cognition, at 32 months (Oudgenoeg-Paz et al., 2015). Mental rotation performance has also been associated with motor competence in 5- to 6-year-olds (Jansen and Heil, 2010). It has also been reported that motor proficiency in childhood is related to extent of physical activity in adolescence (Barnett et al., 2009), and that the development of the strategies required for successful navigation of space is related to cumulative experience of physical exploration of the environment (Cornell et al., 2001). Thus, it seems likely that there is a developmental association between motor abilities and spatial ability beyond that observed in infancy.

One avenue for further investigating the relationship between motor function and spatial cognition is to explore the impact that impaired motor abilities have on spatial cognition. Evidence to-date is sparse, but has demonstrated that adolescents with physical disability show impaired spatial knowledge of their environment (Wiedenbauer and Jansen-Osmann, 2006) and that the extent of this spatial deficit is predicted by their mobility in infancy (concurrent motor ability was not measured) (Stanton et al., 2002). Furthermore, physical activity in children with developmental coordination disorder (DCD) is related to the extent of their motor impairment (Rivilis et al., 2011). Finally, Belmonti et al. (2015) report impaired spatial memory on a table-top task and a large-scale navigation task in children with motor deficits as a result of cerebral palsy. In summary, it is likely that children with motor impairments show delayed exploration of space in infancy, are less physically active, and do not explore their environment as actively as those without motor impairment, and that this has negative consequences for the development of spatial knowledge, in particular, large-scale spatial navigation ability.

Individuals with attention deficit hyperactivity disorder (ADHD), the most prevalent neurodevelopmental disorder during childhood (occurrence: 3–6%) (Polanczyk et al., 2007), present with primary characteristics of hyperactivity, impulsivity, and inattention (American Psychiatric Association [APA], 2013). ADHD is more prevalent in males than females, with a prevalence ratio estimated as between 1:3 and 1:16 females: males (Novik et al., 2006). In addition, a co-occurring motor impairment is evident in children with ADHD, with ∼50% meeting criteria for DCD (Brossard-Racine et al., 2011; Goulardins et al., 2013). There is mixed evidence that motor impairments in ADHD are related to severity of ADHD symptomatology (Kroes et al., 2002; Kopp et al., 2009; Farran et al., submitted). Despite this, it has been shown that the presence of motor deficits in ADHD contributes to poor psychosocial outcome in adults (Rasmussen and Gillberg, 2000). We do not know, however, whether motor deficits are associated with spatial cognition in this group.

Given the association in the typical population between motor competence and spatial cognition in infancy, here we investigate whether this association is evident in TD children into childhood. The studies with infants predominantly investigated large-scale spatial knowledge; this is one reason why we have chosen a large-scale spatial navigation task as the spatial measure for this study. Because little is known about the relationship between motor ability and large-scale spatial knowledge in the typical population beyond infancy, this will add to the body of knowledge surrounding typical development. We will also determine whether the same association between motor ability and spatial ability leads to impaired spatial cognition in those children with ADHD who present with a motor impairment. Therefore, we will explore the developmental relationship between early motor milestones and current motor abilities in children with ADHD, on spatial navigation. To-date spatial navigation has not been investigated in ADHD, and given that poor motor ability in individuals with physical disability is a limiting factor to the development of large-scale spatial knowledge (Stanton et al., 2002), this is our second reason for choosing a spatial navigation task as our measure of spatial ability.

In addition to a comparison of performance in our ADHD sample to that of typically developing (TD) children, we will also compare their performance to the performance of individuals with Williams syndrome (WS). Comparison between ADHD and TD children will determine whether the patterns of performance in the ADHD group are indicative of typical or atypical performance. By using cross-syndrome comparison with WS, we will also be able to differentiate between patterns of performance that are syndrome-specific to ADHD vs. a universal consequence of the presence of a motor deficit. WS is a rare genetic disorder, with an occurrence of 1 in 7,500 to 1 in 20,000, which occurs equally in males and females (Morris and Mervis, 1999; Strømme et al., 2002). Individuals with WS have mild to moderate learning difficulties and an IQ of ∼60 (see Farran and Karmiloff-Smith, 2012). Crucially, we chose WS as our comparison group because it shares deficits with ADHD in attention and motor skill. That is, with reference to attention, Rhodes et al. (2011) report that all of their 19 participants with WS met the criteria for ADHD on the Conners’ Parent Rating Scale (CPRS; Conners et al., 1998). There is also consistent evidence for impaired motor ability in WS. This has been demonstrated with respect to: delayed motor milestones (Carrasco et al., 2005); impairments on standardized motor tasks (Tsai et al., 2008; Atkinson, 2017; Wuang and Tsai, 2017); atypical reaching movements, walking and stair decent (Elliott et al., 2006; Hocking et al., 2010; Cowie et al., 2012). Furthermore, impaired spatial cognition is a hallmark deficit of WS (Farran and Formby, 2012). With reference to large-scale spatial knowledge, impairments are consistently demonstrated in WS (e.g., Farran et al., 2010, 2015; Purser et al., 2015), but the contribution of motor impairments to this deficit is currently unknown. Using WS, we will determine whether different motor deficits (ADHD vs. WS) lead to different patterns of navigation ability and whether specific motor deficits (e.g., fine vs. gross motor) are more detrimental to navigation than others.

In the real world, it is difficult to dissociate the motor and non-motor demands of navigation; concurrent demands of locomotion (e.g., proprioceptive, vestibular demands) cause slow/disrupted movement and disturb effective navigation, making it difficult to uniquely measure spatial knowledge. Here, we will use desktop virtual reality; this neutralizes the inputs from the gross motor system, allowing a purer measure of the spatial aspects of navigation performance, while also maintaining ecological validity. Evidence has also shown that performance in virtual and real-world navigation tasks tap into the same cognitive mechanisms and that learning in a virtual environment (VE) transfers to the real world (Richardson et al., 1999; Coutrot et al., 2019).

The ability to navigate develops through three stages. First, an individual recognizes landmarks within an environment (landmark knowledge). This is followed by knowledge of the relationship between landmarks and turns of a specific route (route knowledge). Finally, configural information of the spatial relationship between landmarks and places within the environment is encoded (configural knowledge or a cognitive map) (Siegel and White, 1975). Note, however, that while these three stages are distinct, it is now considered that they do not necessarily follow a sequential pattern of emergence (see Montello, 1998). Individuals with WS are able to gain both landmark knowledge and route knowledge, but rarely encode a cognitive map of an environment (Farran et al., 2015). This limits their ability to deviate from a fixed learnt route, and thus has an impact on their ability to make short cuts or to reorient when lost.

An associated consequence of less sophisticated navigation skills is a strong reliance on landmarks for effective wayfinding. This is true of individuals with WS, but also young TD children, and thus appears to be a characteristic of immature navigation abilities (Farran et al., 2012, 2016; Lingwood et al., 2015; Purser et al., 2015). Landmarks are objects in the environment that are salient, either perceptually or on account of contextual information (Caduff and Timpf, 2008), and are an important aspect of spatial cognition. For example, in the classic reorientation task, 2-year-olds use landmark information to develop a geometric understanding of a rectangular environment (Learmonth et al., 2001), and we have already discussed the use of landmarks to crawl to a (hidden) target location in infants (Clearfield, 2004).

The ability to select useful landmarks is advantageous during spatial navigation. Landmarks at junctions are more useful than landmarks that are not near a decision point (Farran et al., 2012). Furthermore, proximal landmarks are more useful for developing route knowledge while distant landmarks are more useful for encoding configural information of the environment (Purser et al., 2015). TD children aged from 6 years, and individuals with WS, show stronger recall of landmarks at junctions than landmarks on path segments (Farran et al., 2012). This suggests that both TD children and individuals with WS recognize the usefulness of landmarks at decision points, and support the evidence for a reliance on landmarks for effective route learning. Here we will measure performance on the first two stages of navigation, landmark knowledge and route knowledge. Participants will be asked to learn a fixed route through a novel VE. We will measure the number of errors made while learning the route. Given the importance of landmarks to spatial cognition, and to determine whether participants rely on landmarks to navigate, we will also measure recall of landmarks along the learnt route. These will be divided into landmarks that featured at junctions and landmarks that did not feature at junctions, as an index of the ability to determine landmark usefulness. Alongside navigation performance, we will measure motor skills using a standardized battery of motor ability. In addition, for the atypical groups, we will also obtain parent reports of motor milestone achievement. Given the relationship between environmental factors, such as independent exploration, with motor ability and large-scale spatial knowledge, respectively, we will also measure this environmental factor in our groups.

This is the first study to determine whether the known association between motor and large-scale spatial ability in infancy (see Newcombe, 2019 for a review) extends to childhood and to atypical groups. If motor competence is related to spatial ability, we predict an association between motor ability and spatial ability across all participant groups. Furthermore, we predict that those individuals with a motor deficit (the WS group and a large number of the ADHD group) will show impaired spatial navigation abilities. Of significance, this study will broaden our understanding of the crucial processes that underlay the development of large-scale spatial navigation, with downstream implications for interventions designed to improve navigation performance.

## Materials and Methods

### Participants

Fifty-one children with ADHD (regardless of ADHD subtype) aged 8–15 years were recruited into the study via parent support groups and social media. Three children with ADHD were excluded due to having a co-occurring diagnosis of a neurological condition (partial fetal alcohol syndrome, Tourette’s syndrome, or microcephaly), all of which are associated with problems with movement which could have affected the results. A further two children with ADHD were excluded due to being on medication at the time of testing, which could have positively impacted their motor performance (Kaiser et al., 2015), while three further children with ADHD were excluded because they fell at or below the fifth percentile on our two IQ measures [British Picture Vocabulary Scale III (BPVS), Dunn et al., 2009; Matrices subtest of the British Ability Scales III (BAS), Elliot and Smith, 2011]. One child with ADHD had a co-occurring diagnosis of DCD. This child was not excluded from the analyses. A further 11 children with ADHD with diagnoses of one or more co-occurring disorders were not excluded because ADHD was their primary diagnosis and including these individuals provided a realistic representation of the ADHD population. Furthermore, recent research suggests that ADHD might share common early developmental pathways with other disorders, including autism (see Johnson et al., 2015), and excluding participants with co-occurring disorders would ignore this convergence. These included sensory processing disorder (*N* = 2), pervasive developmental disorder (*N* = 1), dyslexia (*N* = 5), autism (*N* = 3), Asperger’s (*N* = 1), oppositional defiance disorder (*N* = 2), social communication disorder (*N* = 1), and obsessive compulsive disorder (*N* = 1). The final sample consisted of 43 children with ADHD, all of whom had a formal diagnosis of ADHD from a clinician, were medication naïve for at least 24 h prior to testing, had an IQ within the normal range, and received an ADHD index score (Conners’ Parent Rating Scale – Revised Long version; CPRS-R:L; Conners, 1997) which supported their diagnosis of ADHD (≥60).

Twenty participants with WS aged 12–50 years participated in the study. This broad age range is not unusual for this kind of study; this is due to the practical nature of recruiting participants with such a rare disorder, but also because the areas of deficit measured in this study are likely to have plateaued by 12 years (e.g., Farran and Formby, 2012), and thus any within group differences can be accounted for by individual differences rather than developmental factors. All participants with WS had been diagnosed based on phenotypic and genetic information. Genetic diagnosis was based on a fluorescent *in situ* hybridization (FISH) test (see Lenhoff et al., 1997). WS participants were recruited from the records of the Williams Syndrome Foundation, United Kingdom. CPRS-R:L (Conners, 1997) data were also collected for this group to provide an index of whether they displayed ADHD characteristics. Six parents/carers did not complete the questionnaire (Table 1); of the remaining 14 participants, nine received a CPRS-R:L ADHD-index score within the clinical range (≥60) for ADHD.

**Table 1:** Participant details.

	TD (*N* = 71)		WS (*N* = 20)		ADHD (*N* = 43)	
			
	M (SD)	Range	M (SD)	Range	M (SD)	Range
Chronological age (years)	8.410 (1.748)	5.020–11.460	27.619 (8.817)	12.860–50.670	11.403 (1.892)	8.010–15.600
Gender (m/f)	38/33 (53% male)		7/13 (32% male)		35/8 (81% male)	
BPVS-III standard score	103.282 (12.726)	70–128	77.000 (10.079)	70–107	98.302 (11.911)	81–123
BAS-III T-score	49.648 (11.899)	21–79	20.200 (0.696)	20–23	45.067 (12.876)	20–74
BOT2-SF standard score	57.320 (7.487)	41–70	28.500 (4.407)	20–37	43.020 (8.251)	28–65
BOT2-SF raw score	68.450 (9.202)	44–82	43.600 (12.796)	16–69	65.530 (10.110)	38–80
CPRS-R:L ADHD index	NA	NA	67.929 (15.598) (*N* = 14)	47–89	77.814 (7.863)	61–90


*Note: The range of BOT2-SF raw scores of the TD group broadly covers the range of BOT2-SF raw scores of the atypical groups. This is with the exception of one participant with WS who had a BOT2-SF raw score of 16. This person was excluded for developmental trajectory analyses.*

Seventy-two TD children aged 5–11 years participated in the study. The TD sample was recruited from primary schools in the United Kingdom. The age range of the TD children was chosen based on the predicted range of abilities of the neurodevelopmental disorder groups on the motor battery [Bruininks-Oseretsky Test of Motor Proficiency Second Edition Short Form (BOT2-SF)]. One TD child scored below the fifth percentile on the two IQ measures and was excluded from the group, leaving a final TD sample of 71 children. All participants had normal or corrected to normal vision. Participant information is given in Table 1.

### Design and Procedure

Ethical approval was obtained from the UCL Institute of Education Research Ethics Committee (approval number: REC 766; study title: Motor development and navigation in Attention Deficit Hyperactivity Disorder). Following written informed parental consent, the participants were tested individually either at their school, in the research lab, or at the participant’s home. The order of tests was randomized for each participant, and the entire session lasted between 1 h 15 min and 2 h. The battery of tasks included those listed below in addition to two other tasks reported elsewhere (Farran et al., submitted).

### Background Tasks

All participants completed the Matrices subtest of the BAS (Elliot and Smith, 2011), and the BPVS (Dunn et al., 2009) as measures of IQ. Standard scores for these tests are presented in Table 1. Standard scores for the BPVS III have a mean of 100 and a standard deviation of 15, while standard scores for the BAS III have a mean of 50 and a standard deviation of 10. In addition, parents/carers of the atypical groups completed the Long Form of the CPRS-R:L in order to derive ADHD index scores. Scores on subscales that are one standard deviation above the mean of 50 (i.e., scores of 60 or above) are considered to be in the clinical range. The test–retest reliability for the ADHD index is 0.72.

### Motor Task: Bruininks-Oseretsky Test of Motor Proficiency Second Edition Short Form (Bruininks and Bruininks, 2005)

The BOT2-SF is a measure motor competence for individuals from 4 to 21 years. Raw composite scores and standard scores for this test are presented in Table 1; standard scores have a mean of 50 and a standard deviation of 10. The composite score is the sum of performance on eight subtests (comprised from 14 items). The fine motor control subtests are: Fine Motor Precision, Fine Motor Integration, and Manual Dexterity. The gross motor control subtests are: Bilateral Coordination, Balance, Running Speed and Agility, Upper Limb Coordination, and Strength. In addition to a Fine Motor and a Gross Motor score, a combined Motor Composite score can also be derived. The Short Form range has good test–retest reliability (0.80–0.87) and interrater reliability (0.98).

### Motor Milestones Questionnaire

A parental questionnaire (developed by Sumner et al., 2016, which was based on Brouwer et al., 2006) was used to investigate the extent to which the children with ADHD and individuals with WS reached motor milestones. Parents were asked to give the age (in months) that 12 significant milestones were reached, six of which have been standardized against World Health Organization (WHO) data (WHO Multicentre Growth Reference Study Group, 2006). The data from these six milestones only are reported here (for details of the results of the full questionnaire, see Farran et al., submitted).

### Environmental Measure: Independent Exploration (Based on Shaw et al., 2015)

A short questionnaire was read aloud to the individual and on occasion their parent to obtain information of experience of exploration. Participants were asked questions regarding the extent to which they were allowed to explore environments with others or by themselves. For example, they were asked about how and with whom they got to and from school and if they independently went to local shops or the park. They were also asked about the frequency of these behaviors. A composite exploration score was determined based on the sum of five independent activities and the frequency at which these activities took place in a typical week (max total score: 31). A binomial score was also calculated from this which determined whether the participant was permitted to explore independently (composite score ≥ 1 = binomial score 1) or not (score = 0).

### Large-Scale Spatial Navigation

**FIGURE 1:**
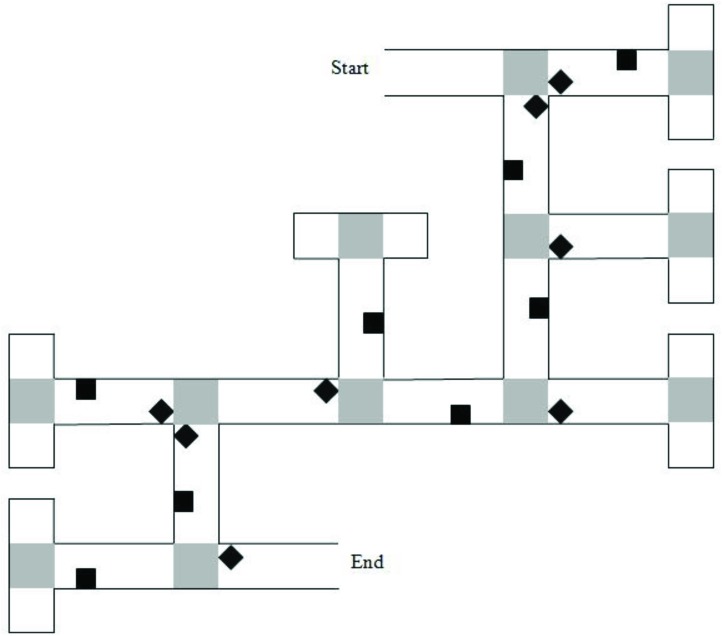
Map of the six-turn maze layout. Gray squares represent “pebble” texture that was featured at junctions and at the end of cul-de-sacs. Black diamonds indicate junction landmarks. Black squares indicate path landmarks. Reprinted from Farran et al. (2012), with permission from Elsevier.

Two VEs were created using Vizard^1^ and presented on a 17-in laptop computer. The VEs displayed mazes with either six or eight junctions/decision points. Each junction led to two paths, one correct and one incorrect. Incorrect path choices ended in a cul-de-sac, which had the same appearance as a T-junction when viewed from the preceding junction. Mazes were lined with brick walls and landmarks (objects) were placed on both correct and incorrect sections of the route (Figure 1). Landmarks were selected from a range of categories (e.g., animals, tools, furniture) for their high verbal frequency (Morrison et al., 1997) and for being easy to recognize. Landmarks within the maze were equally distributed to the left and right of the path. At the end of the maze was a gray duck, which once approached, ended the game.

The six-junction route had been previously used by Farran et al. (2012). It had 16 unique landmarks. Eight of the landmarks were near to junctions (“junction landmarks”). Eight of the landmarks were not near to junctions (“path landmarks”). Across these junctions, there were two left, two right, and two straight-ahead choices that led to the next correct path segment. A map of the maze layout is shown in Figure 1. The eight-junction route was created for this study. It had 20 unique landmarks; 10-junction landmarks and 10-path landmarks. Across the junctions, there were three left, three right, and two straight-ahead choices that led to the next correct path segment.

#### Corridor Task

Preceding the experimental mazes, participants were given the opportunity to practice navigating along a simple corridor which did not include decision points or landmark objects, but included two turns. Participants were instructed on how to navigate the VEs by using the four arrow keys on the keyboard. They then watched the experimenter navigate the corridor, before navigating it themselves. This involved simply following the path, which included two right-angle turns; there were no decisions to be made. If participants had difficulty controlling their navigation, they were given another walk of the corridor. No participants required more than two walks of the corridor.

#### Route Learning Task (Six-Junction Route)

The experimenter showed the participant the correct route through a six-junction maze. The experimenter instructed the participant to “Pay close attention to the route and to the objects that appear in the ‘maze game’ because you will have to go exactly the same way through the maze after I have shown you.” After the experimenter had demonstrated the correct route, the participants attempted to walk the correct route from start to finish using the arrow keys. If an incorrect path was selected, participants reached a cul-de-sac and were able to self-correct by turning around. Encouragement was given, but no help. If a participant turned toward the start of the maze, they were directed back to the junction where they made the error. Each trial terminated on reaching the gray duck and completing the route. Each walk through the maze from start to finish was labeled as a learning trial. Participants completed learning trials to a criterion of completing the maze from start to finish without error on two consecutive trials, or until they had completed 10 learning trials. The cumulative number of errors across learning trials was recorded. An error was defined as a deliberate incursion down an incorrect path; if the participant corrected his/her course before reaching half-way down an incorrect path section, no error was counted.

#### Landmark Recall Task

**FIGURE 2:**
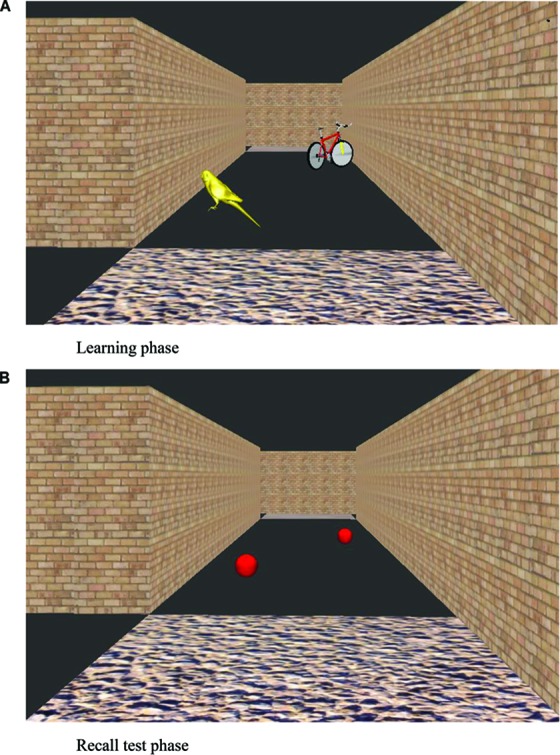
View of a virtual environment during learning trials **(A)** and at the recall test phase **(B)**.

After the participant had learnt the six-junction route to criteria, the landmark recall task commenced. The experimenter showed the participant the same maze but with all landmark objects shown as red balls (Figure 2). The experimenter navigated the route themselves and stopped at each junction to point out each red ball in the subsequent path section. Participants were asked what object the ball had been when they were walking around the maze. After an answer was given, the participants were then shown an image of the landmark object in its correct location, on another computer screen. This was conducted for all landmark objects that were visible from the correct path (eight landmarks on the correct path in addition to four landmarks that featured on incorrect path sections that could be viewed straight ahead before a correct turn to the left or right was executed). Landmark recall score was calculated as the number of correctly identified junction and path landmarks that featured at junctions (Max. = 6 for each landmark category, junction and path landmarks).

#### Naming Task

A naming task was administered after the landmark recall task, to ensure that the verbal labels used by the participants in the landmark recall task could be coded accurately (e.g., a participant might use the word “light” for “streetlamp”). Participants were shown images of each of the 16 landmarks in a pseudo-random order and were asked to name them. Participants received a score out of 16 on the naming task.

#### Route Learning Task (Eight-Junction Route)

Following the six-junction maze, in order to ensure a wide range of variability in route knowledge performance, participants were shown a longer eight-junction route in a different VE, and asked to walk the route themselves using the same procedure as used for the six-junction maze. For this route, participants simply completed three trials and the cumulative number of errors was recorded. No landmark recall task or naming task was completed. To determine overall maze error score, the cumulative number of errors across all learning trials in the six-junction maze and the three learning trials in the eight-junction maze was calculated for each participant.

## Results

Note that there were 11 children with ADHD who had co-occurring diagnoses that we had no reason to believe would impact the pattern of results. To exercise caution, all analyses listed below were run a second time, with these 11 children excluded. This only changed one result with respect to motor milestone data (noted in the manuscript). As such, results are presented with these children included in the analyses.

We are specifically interested in whether an impairment in motor competence has an impact on large-scale spatial knowledge. Given that large-scale spatial knowledge is associated with independent exploration (Cornell et al., 2001), we are also interested in whether poorer motor ability is associated with reduced independent exploration, and in turn, large-scale spatial knowledge. To this end, we have chosen two groups who are known to have motor impairments, ADHD and WS, the latter of which also presents with spatial deficits. The TD group was chosen to span the range of motor abilities of the ADHD and WS group in order that developmental trajectory analysis could be carried out on large-scale spatial performance, with motor ability as a measure of motor “mental age” (Thomas et al., 2009). Before detailing performance on the spatial navigation task, we first present the motor and independent exploration demographics of each group. Each participant completed the BOT2-SF and the independent exploration questionnaire, while the parents of the ADHD and WS samples also completed a motor milestone questionnaire.

### Motor Performance: BOT2-SF

The BOT-SF is a standardized measure with standardized scores classified as falling within a number of zones. The TD sample fell within the “average” (*N* = 43), “above average” (*N* = 25), and “well above average” (*N* = 3) zones, indicative of no motor impairment. The ADHD group fell within the “well below average” (*N* = 2), “below average” (*N* = 18), “average” (*N* = 22), and “above average” (*N* = 1) zones; this indicates that 20 of the 43 participants with ADHD presented with a motor impairment (≤16th percentile). The WS group fell within the “below average” (*N* = 7) and “well below average” (*N* = 13) zones and thus all presented with a motor impairment.

### Motor Milestones

Motor milestone data were collected for ADHD and WS groups only. Data are presented for six motor milestones, and compared to percentiles based on WHO data (WHO Multicentre Growth Reference Study Group, 2006) in Table 2. Note that due missing data, the data presented are based on reduced Ns (Ns for each milestone for each group are given in Table 2).

**Table 2:** Motor milestone month of achievement for the WS, ADHD-L, and ADHD-H groups compared to typical month of achievement.

	WHO Age in months at which milestone achieved		WS				ADHD			
			
	*M* (SD)	Range	*N*	*M*(SD)	P’tile	Range	*N*	*M*(SD)	P’tile	Range
Sit without support	6.0 (1.1)	3.8–9.2	9	12.222 (5.911)	>99th	3–24	30	6.100 (1.589)	50th	3–10
Crawl hands and knee	8.5 (1.7)	5.2–11.4	6	15.500 (5.612)	>99th	5–21	28	8.607 (2.254)	50th	3–13
Stand with assistance	7.6 (1.4)	4.8–11.4	7	16.286 (6.130)	>99th	10–24	31	9.000 (2.758)	90th	3–18
Stand without support	11.0 (1.9)	6.9–16.9	5	24.600 (9.370)	>99th	12–36	33	11.136 (2.356)	50th	7–19
Walk with assistance	9.2 (1.5)	6–13.7	6	21.167 (9.131)	>99th	12–36	33	11.288 (2.414)	90th	6–19
Walk without support	12.1 (1.8)	8.2–17.6	13	24.615 (8.921)	>99th	15–42	36	13.125 (2.831)	75th	9–24


*WHO = World Health Organization. WHO Milestones data from the WHO Multicentre Growth Reference Study Group (2006). Note: Ns differ across cells due to missing data.*

Contrary to the findings from the BOT2-SF above, overall the ADHD group achieved motor milestones broadly within the typical range of achievement, with a slightly wider range of achievement than for the typical population. The WS group achieved all six motor milestones later than would be expected for a TD child (although note that the range of month of achievement for the WS group overlaps with the typical range). Motor milestone achievement was not related to concurrent motor ability (BOT2 Overall score) for either group [*p* > 0.008 for all (Bonferroni corrected alpha)]. Although note that when the 11 ADHD participants with comorbid conditions were excluded, age of independent walking correlated with both BOT2-SF fine motor score (*p* = 0.007) and the residuals (age partialled out) (*p* = 0.002) of this measure for this group.

### Independent Exploration

An exploration score was not available for one TD participant (and thus *N* = 70 for the TD group). Descriptive statistics are presented in Table 3. Exploration score was related to age for the TD group [Spearman’s rho (70) = 0.635, *p* < 0.001] and thus the TD group could be used as a method of standardization for the atypical groups. To do this, the TD group was split into three age groups [TD 5–6 years (*N* = 20), TD 7–8 years (*N* = 20), TD 9–11 years (*N* = 30)] for comparison with the atypical groups. Note that the TD 9–11-year-old group also did not differ from the ADHD group for chronological age and thus represented an age-matched comparison group (ADHD: *p* = 0.530). The data for the TD 5–6-year-olds, the TD 7–8-year-olds, and the WS group were not normally distributed due to a large number of zero scores (Kolmogorov–Smirnoff test, *p* < 0.05 for all). As such, a Friedman ANOVA was carried out with group as a between participants factor. This demonstrated a main effect of group, χ^2^(4) = 35.732, *p* < 0.001. Mann–Whitney-U paired comparisons demonstrated that the WS group explored to a greater extent than the TD 5–6-year-olds (*p* < 0.001), but at a similar level to the 7–8-year-olds (*p* = 0.284) and 9–10-year-olds (*p* = 0.270). The ADHD group was exploring more than the TD 5–6-year-olds (*p* < 0.001) and the TD 7–8-year-olds (*p* = 0.002), but at the same level as the 9–11-year-olds (*p* = 0.723). Thus, the ADHD groups were exploring at the level appropriate for their chronological age, while the WS groups were exploring at the level of a 7–11-year-old child, even though they were adults.

**Table 3:** Exploration scores for each participant group.

	TD 5–6 (*N* = 20)		TD 7–8 (*N* = 20)		TD 9–11 (*N* = 30)		WS (*N* = 20)		ADHD (*N* = 43)	
					
	Median	Range	Median	Range	Median	Range	Median	Range	Median	Range
Exploration score (Max:31)	0	0–9	3	0–21	6.5	0–21	3.5	0–22	7.0	0–23
% Permitted to explore	10.0%		57.1%		90.0%		80.0%		86.0%	


The relationship between exploration score and BOT2-SF overall motor score was determined using Spearman correlations. Because age was related to exploration score for the TD and ADHD group (*p* < 0.05 for both), the residuals of exploration score (age partialled out) were also used for these two groups to determine the relationship after accounting for age-related variance. This demonstrated a relationship between exploration score and motor ability for the TD and ADHD groups only (TD: *p* < 0.001; ADHD: *p* = 0.014; WS: *p* = 0.112), which was accounted for by variance in age (TD: *p* = 0.131; ADHD: *p* = 0.223).

Due to the large number of zero scores, a binomial score was also calculated which determined whether the participant was permitted to explore independently or not. The percentage of participants who received a score of 1 (i.e., they were permitted to explore independently) is also shown in Table 3.

### Spatial Navigation

Two primary dependent variables were derived from the navigation task, maze error score and landmark recall. Maze error score is a measure of an individual’s ability to learn a route, i.e., route knowledge. Landmark recall score provides information about strategy use when learning the route, i.e., did participants use landmarks as an aid to learning the route, and were landmarks at junctions considered strategically more useful than landmarks on paths?

Spatial navigation was analyzed with respect to variation in motor competence across our participants using developmental trajectory analysis. Developmental trajectory analysis is used to ascertain whether the trajectory of performance across the range of mental ages (in this case motor mental age) of each group differs in: mean value; intercept; or slope (rate of development). To determine which measures of motor ability were most suitable as a measure of motor “mental age,” correlational analyses were carried out for each group between the two spatial measures, maze errors and landmark recall (for all 12 landmarks) and five motor measures [BOT-2 gross motor score, BOT2 fine motor score, walking unsupported (atypical groups only), hands and knees crawling (atypical groups only), exploration score] (Table 4). On account of significant input from chronological age to BOT-2-SF gross and fine motor scores and exploration scores for the TD and ADHD groups (*p* < 0.05 for all), correlations were also included for the residuals of these three measures for these two groups (age partialled out). This constituted up to 16 correlations per atypical group and 12 correlations for the TD group, thus we used Bonferroni corrected critical alphas (atypical groups: *p* ≤ 0.003; typical group: *p* ≤ 0.004). Due to the very small sample size for crawling for the WS group (*N* = 6), these correlations would not be informative and so are not reported.

**Table 4:** Bivariate correlations with maze error and landmark recall.

Group		BOT-2 raw motor score				Motor milestones		Exploration	
				
		Raw		Residuals (age partialled out)				Raw	Residuals (age partialled out)
					
		Gross	Fine	Gross	Fine	Crawling	Walking		
TD	Maze error	-0.332	-0.481^*^	-0.172	-0.395	NA	NA	-0.034 (*N* = 70)	0.145 (*N* = 70)
	Landmark recall	-0.061	-0.027	-0.096	-0.029			-0.095	-0.106
WS	Maze error	0.118	-0.163	NA	NA	NA	-0.203 (*N* = 13)	-0.157	NA
	Landmark recall	-0.063	0.399				-0.054 (*N* = 13)	0.184	
ADHD	Maze error	-0.308	-0.364	-0.186	-0.192	0.027	0.079	0.169	-0.359
	Landmark recall	0.123	0.151	0.075	0.097	-0.110	0.027	0.131	0.181


*^∗^p ≤ 0.004 (TD critical alpha); No correlations met the disorder group critical alphas of p ≤ 0.003. Note: Ns are reported where the data was not available for the full sample.*

### Associations Between Spatial Navigation and Motor Performance

None of the motor scores or exploration score correlated with *landmark recall* for any of the groups (*p* > 0.003 for all). Despite medium effect sizes for the BOT2-SF measures for the ADHD group (Table 4), there were no (Bonferroni corrected) significant correlations with *maze error* for the ADHD and WS groups (*p* > 0.003 for all). Maze error correlated with BOT2-SF fine motor scores for the TD group (*p* ≤ 0.004; Gross motor score: *p* = 0.005). Correlations with the residuals demonstrated that any association between BOT2-SF gross motor score and maze error in the TD group was mediated by age, *r*(71) = -0.179, *p* = 0.135. This was not the case for BOT2-SF Fine motor score, *r*(71) = -0.395, *p* = 0.001.

### Maze Error Score

As shown in Table 4, BOT2-SF fine motor ability demonstrated a small (*r* = 0.10) to medium (*r* = 0.30) effect size (Cohen, 1992) for all groups for maze error score, albeit only to (Bonferroni corrected) significance for the TD group. As such, BOT2-SF fine motor ability was deemed the best measure of “mental age” for developmental trajectory analysis (Thomas et al., 2009). Developmental trajectory analysis can be influenced by outliers, thus we used an exclusion criteria of maze error scores that were three standard deviations above the group mean. One participant in the ADHD-L group met this exclusion criteria only [this changed the correlation reported in Table 4 to *r*(42) = -0.164]. In order for the ranges of the covariates to be largely overlapping, one WS participant who only achieved a fine motor score of 1 was excluded [this changed the correlation reported in Table 4 to *r*(19) = -0.252]. In order that any differences in intercepts were meaningful, BOT2-SF fine motor score was rescaled such that the intercept was at the lowest BOT2-SF fine motor score of the participants. This does not change the analysis, but enables meaningful interpretation of the intercept.

Initial ANOVA of group means revealed that maze error differed across groups, *F*(2,129) = 17.288, *p* < 0.001, η*_p_*^2^ = 0.211. Tukey paired comparison demonstrated that this was due to higher maze error score in the WS group relative to all other groups (*p* < 0.001 for both), with no differences across the remaining groups (*p* > 0.05). ANCOVA with BOT2 fine motor as a covariate demonstrated a significant impact of BOT2 fine motor score [*F*(1,126) = 10.541, *p* = 0.001, η*_p_*^2^ = 0.079]. There was also a significant group difference in the intercept of maze error scores [*F*(2,126) = 4.304, *p* = 0.016, η*_p_*^2^ = 0.064], such that at the lowest motor ability, the ADHD group had lower maze error scores than the TD and WS groups (ADHD and WS: *p* = 0.036; ADHD and TD: *p* = 0.003; TD and WS: *p* = 0.281). Note that this difference in intercept remained when BOT2-SF fine motor score was replaced by the residuals (age partialled out) of this variable (*p* = 0.024). The slope of the relationship between motor ability and maze error score did not differ across groups [*F*(2,126) = 1.85166, *p* = 0.159, η*_p_*^2^ = 0.029].

#### Naming Score

Participant’s naming scores were sufficiently high that we could be confident that all participants were able to provide verbal labels for the landmarks [mean (SD) out of 16: TD: 15.521 (0.790); WS: 15.200 (1.001); ADHD: 15.628 (0.757)], thus enabling accurate scoring of landmark recall. Naming score was consistent across groups, *F*(2,131) = 1.904, *p* = 0.153, η*_p_*^2^ = 0.028. Where participants named the item inaccurately (e.g., “jelly” for “cake,” or “bat” for “tennis racket”), we accepted this answer in the landmark recall task as accurate.

#### Landmark Recall

**FIGURE 3:**
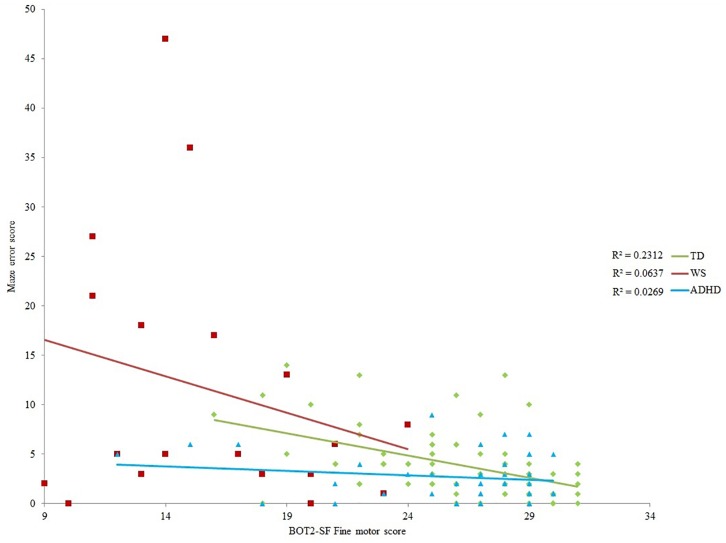
The relationship between maze errors and motor ability (BOT2-SF fine motor), by group.

As observed in Table 4, effect sizes were often below the cut-off for a small effect, which indicates that motor ability was not related to landmark recall. As such, it was not possible to carry out developmental trajectory analysis. Landmark recall was also not related to chronological age (*p* > 0.05 for all groups). Consequently, landmark recall was analyzed using ANOVA with a between-participant factor of Group (TD, ADHD, WS) and a repeated measures factor of landmark type (junction landmarks, path landmarks). There was a main effect of group, *F*(2,131) = 3.413, *p* = 0.036, η*_p_*^2^ = 0.050, due to poorer landmark recall in the WS group, compared to the TD group (*p* = 0.043) only (all other *p*’s > 0.05). The effect of landmark type enables us to draw conclusions about strategy use in each group. As shown in Figure 3, there was a main effect of landmark type, *F*(1,131) = 39.300, *p* < 0.001, η*_p_*^2^ = 0.231, due to weaker recall of path landmarks than junction landmarks. This effect did not interact with group, *F* < 1, which indicates consistent use of landmarks to learn the route across all groups. To further determine whether the use of a landmark strategy was associated with success at learning routes, we investigated the relationship between landmark recall score and maze error score for each group. This demonstrated that maze error score was not related to landmark recall score for any group: TD: *r*(71) = -0.029, *p* = 0.808; WS: *r*(20) = -0.409, *p* = 0.073; ADHD: *r*(42) = -0.274, *p* = 0.075.

## Discussion

In this study, we demonstrated that the relationship between motor competence and large-scale spatial cognition observed in infancy (Clearfield, 2004) is also observed in TD children aged 5–11 years. This contrasted to no relationship between motor competence and large-scale spatial cognition in children with ADHD or individuals with WS. Furthermore, while the WS group demonstrated impairments in both the motor and spatial domains, the ADHD group did not show any deficits in large-scale spatial cognition, despite evidence of impairment in the motor domain. We suggests that a motor impairment does not necessarily lead to a deficit in large-scale spatial cognition, and that spatial ability can develop independent of the motor domain.

On account of the novelty of our dataset within the TD literature, we first discuss the novel findings with respect to the TD group only, before comparison across the TD, ADHD-L, ADHD-H, and WS groups.

### Typical Development

The findings from the spatial navigation task replicate previous studies (e.g., Farran et al., 2012). That is, all TD children were reliant on landmarks to remember the route as evidenced by stronger memory for landmarks that featured at junctions (i.e., more useful landmarks) compared to landmarks that featured on path sections.

Having demonstrated successful spatial navigation in the TD children, we were interested in how motor ability related to this ability. We found that, at least for some aspects of motor ability, there is a relationship between performance in the motor and spatial domains. That is, for TD children aged 5–11 years, stronger fine motor ability is associated with fewer errors on a route learning task (23% of variance explained), even after controlling for variation in age. The relationship between gross motor ability and spatial ability, however, simply reflected age-related variation. Spatial ability in infancy has been assessed in relation to the development of both gross motor milestones (e.g., Clearfield, 2004) and fine motor skills (Soska et al., 2010), both reporting an impact of motor skill on spatial understanding. Similarly, motor ability is related to mental rotation ability in 5- to 6-year-olds (Jansen and Heil, 2010). Our findings support and extend the findings of Jansen and Heil (2010) by demonstrating that the relationship between motor ability and spatial cognition in infancy is evident across the primary school years with respect to large-scale spatial cognition.

The interpretation that has been put forward for the association between motor and spatial abilities in infancy relates predominantly to the development of self-movement either through crawling or walking; with the new ability to move comes the requirement for the infant to focus their attention on their spatial environment, which has a positive impact on spatial cognition (Clearfield, 2004). Soska et al. (2010) further our understanding of the importance of new attentional perspectives; they explain that fine motor skills such as transferring an object from hand to hand and rotating an object while looking at it, enables the infant to learn about objects from different viewpoints. This leads an infant to understand the three-dimensional nature of objects. The association observed in this study in relation to fine motor ability in childhood expands our understanding of this cross-domain relationship.

Oudgenoeg-Paz and Rivière (2014) situate the motor-spatial relationship within the theory of embodied cognition. They explain that sensory-motor interaction with the environment facilitates spatial development. Our finding of a relationship between fine motor ability and spatial ability supports an embodied cognition explanation. It is likely that the association observed in 5–11-year-olds is not a direct consequence of a step change in awareness of the spatial environment (as in infants), but represents the continuation of the relationship observed by Soska et al. (2010) in infancy. That is, it is a result of increased understanding of space via physical manipulation of objects and manipulation of the relationships between objects within the environment, which requires fine motor skills. We suggest that this likely benefits skills such as the ability to perform mental transformations and perspective taking, both of which are spatial skills that feed into navigation performance (Broadbent, 2015). In summary, this is the first study to demonstrate the importance of motor ability for large-scale spatial cognition, in the typical population, beyond infancy.

Children were asked about their independent exploration, such as whether they were allowed to walk home from school alone or to cross roads alone. While only 10% of TD 5–6-year-olds indicated that they had performed at least one of these independent acts in the preceding week, 60% of TD 7–8-year-olds reported independent exploration. This contrasts to 5–10% of children aged 7–8 years reported by Shaw et al. (2015) and hence questions the reliability of the self-reports of the children in the TD group (although note that the schools in this study were in inner London and so a local shop might be relatively close in comparison to other locations). Shaw et al. (2015) used parent report with 512 parents of the United Kingdom 7- to 15-year-olds. Despite this, we did see the anticipated relationship between increasing independent exploration and age, which supports the validity of the measure. Exploration score, did not, however, relate to motor ability, or to either of the spatial measures (maze error score or landmark recall score). This does not support the embodied cognition notion that motor action enables exploration, which in turn impacts cognition (Smith and Gasser, 2005). It also contrasts with Cornell et al. (2001) who demonstrated a relationship between exploration of the local environment and spatial navigation ability. In addition to the potential limitation in reliability mentioned above, it is possible that the exploration measure did not capture the kind of exploration that is employed by this age range. Independent exploration outside of the home is relatively limited for UK children due to cultural and safety reasons (Shaw et al., 2015). Perhaps a measure adapted from Oudgenoeg-Paz et al. (2015) in which exploration is measured in a safe environment would provide a more sensitive and reliable measure of this variable.

### Neurodevelopmental Disordered Groups

The primary aim of the cross-syndrome comparison between individuals with ADHD and individuals with WS was to determine whether the presence of a motor deficit dictates that (large-scale) spatial cognition will also be impaired. This was based on the known relationship between the achievement of motor milestones and spatial abilities in the typical population, as well as findings that physical disability can negatively impact large-scale spatial knowledge (Stanton et al., 2002). The findings from the TD group in this study, discussed above, also demonstrate a relationship between motor ability and spatial cognition, in children aged 5–11 years.

**FIGURE 4:**
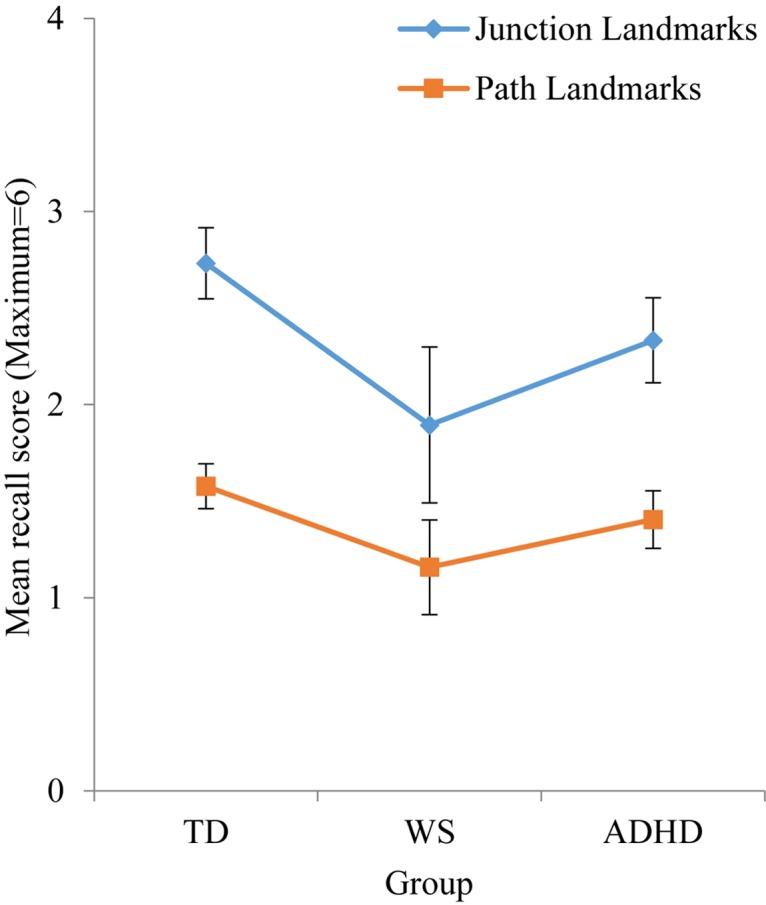
Mean numbers of junction and path landmarks correctly recalled during test phase. Error bars represent standard error.

If a motor deficit has a cascading downstream negative impact on spatial abilities, then the poorest spatial navigation performance should have been observed in those with a motor impairment (the WS group, and approximately half of the ADHD group, i.e., those ADHD participants on the left half of Figure 4). This prediction was not borne out. In fact, at the lowest level of motor ability (the intercept), the ADHD group had statistically *lower* maze errors than the TD group—although this finding must be interpreted with caution due to the low amount of variance in route learning errors explained by motor ability in this group. Motor ability explained 6% (WS) and 3% (ADHD) of variance in route learning errors. This demonstrates that motor competence is not a significant contributor to large-scale spatial ability for these groups. Furthermore, only the WS group demonstrated a deficit in spatial navigation. Spatial navigation in the ADHD group was on a par with than that of the TD group, which indicates that there is no large-scale spatial impairment in this group. This cross-syndrome difference between the WS and ADHD groups, coupled with the lack of significant association between motor and spatial competence across both of the disorder groups suggests that, in contrast to the spatial deficits observed in children with physical disability (Stanton et al., 2002), a motor impairment need not lead to an impairment in large-scale spatial cognition. This is, however, within the context of a small sample size for the WS group. Nevertheless, the effect sizes presented in Table 4 do not suggest that any non-significance relates to lack of power in this sample.

The above finding has two possible interpretations. First, perhaps motor milestone achievement plays a larger role in the development of spatial cognition, than later motor competence. That is, if motor milestones are achieved late, then this could be critical for the development of early spatial ability, with cascading negative impact on the development of the spatial domain. Note that the parents/carers of the WS group report delayed motor milestone achievement of their children, but the parents/carers of the ADHD group report that their children achieve motor milestones at a broadly typical time, regardless of their concurrent motor ability. There was a hint that the age of walking onset in ADHD is related to concurrent motor competence, as this was a significant association when the children with comorbid diagnoses were excluded. It is possible that the motor difficulties experienced by some children with ADHD stem from more subtle motor (or attention) deficits in infancy (see Farran et al., submitted for further discussion), which were not measured here. This requires further investigation using more sensitive measures, such as investigation of motor quality of walking in this group. Nonetheless, walking was not achieved substantially later than TD children in this group. This contrast between WS and ADHD groups with respect to motor milestones mirrors the pattern of impaired spatial ability in the WS group, but not the ADHD group. This is also consistent with delayed motor milestone achievement in children with physical disability, who also demonstrate impaired navigation performance (Stanton et al., 2002). Despite this, motor milestone achievement was not significantly related to spatial ability in either the ADHD or WS group. Of course, this could be due to a lack of power (there was missing data for this measure). Furthermore, although this kind of retrospective report has been shown to be as reliable as concurrent assessments (Langendonk et al., 2007), it is possible that the retrospective nature of this measure impacted reliability in our sample, particularly given that many of the WS group were adults. Further data are therefore required to support or refute this hypothesis.

The second possible interpretation is that, while a relationship is observed between motor ability and spatial ability in both infancy (Clearfield, 2004), and TD children (Jansen and Heil, 2010, this study), motor competence might not be a prerequisite for the development of large-scale spatial competence. That is, if the usual developmental pathway is limited, then over developmental time, it is possible that large-scale spatial skill development is redirected to alternative pathways, i.e., a pathway which is less reliant on input from the motor system and more reliant on other mechanism that are important to spatial navigation (spatial, memory, and executive function mechanisms). This has been observed for the language domain, where individuals with WS demonstrate language acquisition before the use of joint attention, an ability which was initially thought to be a prerequisite for the acquisition of language (Laing et al., 2002). If spatial ability can develop without input from the motor system, this suggests that the motor impairment and the spatial impairment observed in WS are unrelated, and also explains why the ADHD group demonstrates a large range of motor abilities, but typical large-scale spatial abilities. That is, poor motor competence in approximately half of the ADHD sample and all of the WS group was not a limiting factor to the development of spatial navigation abilities, and the disparity in spatial ability between these two groups was independent of their motor ability. To further support this hypothesis, it would be interesting to employ a wider battery of both small-scale and large-scale spatial tasks, and to investigate this relationship longitudinally, from infancy, in these groups.

This is the first investigation of large-scale route knowledge in individuals with ADHD. This group demonstrated typical route knowledge, i.e., the ability to learn a route from A to B. Of interest, both of the neurodevelopmental disorder groups employed the same, typical strategy to remember the route. That is, they used landmarks to determine which way to turn. However, despite the use of a typical strategy, the WS group recalled fewer landmarks overall and took longer (more errors and hence more trials) to learn the route than the other groups, and performed at the level below a typical 5–6-year-old. This is broadly consistent with previous research and reflects their hallmark deficit in spatial cognition (e.g., Farran et al., 2012; Purser et al., 2015).

While we used a relatively pure spatial navigation task by design, it is also entirely possible that children with ADHD might experience navigation difficulties on account of the attentional and sensory integration of additional demands that are present in real-world navigation (locomotion demands, proprioceptive and auditory information, a richer visual array). This is unlikely given that VEs have been shown to tap into the same cognitive mechanisms as real-world environments (Coutrot et al., 2019). Nonetheless, a deficit in real world, but not virtual navigation, in ADHD, would point toward difficulties in integrating information rather than a purely spatial deficit.

We included an environmental measure that might have impacted large-scale spatial knowledge in our groups, independent exploration. This did not demonstrate a relationship with large-scale navigation performance in either of the groups, perhaps due to the impact of non-motor variables related to dangers in the outside world which might have limited participants’ opportunity to explore. As discussed earlier, a “safe” measure of exploration might have been more sensitive. Comparison across the groups showed only subtle, albeit significant, differences. The WS group showed an exploration at the level of 7–11-year-olds, despite being adults. It is likely that independent exploration is restricted in WS due to their low IQ and hypersociability, which make them particularly vulnerable (Farran and Karmiloff-Smith, 2012). The ADHD group explored at the level of 9–11-year-olds, and thus at a level commensurate with their chronological age.

In summary, we investigated spatial navigation in ADHD for the first time. This demonstrated a typical level and pattern of abilities in this group, which was not impacted by whether the individual displayed a motor impairment or not. Furthermore, cross-syndrome comparison between ADHD and WS demonstrated that a motor impairment in these groups is not associated with large-scale spatial navigation ability. Finally, although our data suggest that the timepoint of motor milestone achievement does not impact the development of large-scale spatial abilities, this conclusion is given with caution due to the large amount of missing motor milestone data in our sample. Indeed, our findings contrast with those of Stanton et al. (2002) who demonstrated a relationship between motor ability in infancy and large-scale spatial navigation in individuals with physical disability.

## Conclusion

This is the first study to demonstrate that the relationship between motor ability and large-scale spatial cognition observed in typical infants (Clearfield, 2004) extends to TD children aged 5–11 years. This supports an embodied cognition view of development and suggests that this cross-domain relationship is present across the primary school years. In contrast, motor ability and large-scale spatial ability were not related in any of the neurodevelopmental disorder groups. This suggests that a motor impairment does not necessarily lead to a deficit in large-scale spatial cognition, i.e., spatial ability *can* develop via an alternative developmental pathway, with little or no input from the motor domain. With respect to each group, in the first study to measure large-scale spatial ability in ADHD, we demonstrated that despite a motor impairment, the children with ADHD and low motor ability displayed competent, age-appropriate, navigation abilities. Furthermore, we measured two of the most impaired domains in WS, motor ability and spatial ability, within the same study for the first time; our findings demonstrated that these two deficits are unrelated in this group. Knowledge that the developmental pathway for spatial cognition is atypical in WS has implications for how best to train navigation abilities to improve independence in this group.

## Data Availability

The datasets generated for this study are available on request to the corresponding author.

## Ethics Statement

Ethical approval was obtained from the UCL Institute of Education Research Ethics Committee (approval number: REC 766; study title: Motor development and navigation in Attention Deficit Hyperactivity Disorder). Following parental consent, the participants were tested individually either at their school, in the research lab, or at the participant’s home.

## Author Contributions

EF conceived of the study, with input from AK-S and EH. AB, HD’S, and LM collected and coded the data. EF analyzed the data and wrote the manuscript with input from EH, AB, HD’S, and LM.

## Conflict of Interest Statement

The authors declare that the research was conducted in the absence of any commercial or financial relationships that could be construed as a potential conflict of interest.

## Footnotes

1 http://www.worldviz.com
